# Use of a fully closed-loop ventilation mode in long-term ventilated ICU patients: a prospective study

**DOI:** 10.1186/cc10722

**Published:** 2012-03-20

**Authors:** J Arnal, A Garnero, M Wysocki, D Demory, G Corno, A Berric, S Donati, L Ducros, J Durand-Gasselin

**Affiliations:** 1 Hôpital Ste Musse, Toulon, France; 2 Hamilton Medical, Bonaduz, Switzerland

## Introduction

IntelliVent-ASV^® ^is a closed-loop ventilation mode that automatically adjusts ventilation and oxygenation settings in passive and active breathing patients. The minute volume is adjusted according to end-tidal CO_2 _(ETCO_2_) information in passive breathing patients (and respiratory rate in active breathing patients), and oxygenation is adjusted according to SpO_2 _information. This study reports the ventilation and oxygenation delivered by IntelliVent-ASV^® ^in long-term ventilated ICU patients.

## Methods

This prospective, observational study included 100 patients invasively ventilated using IntelliVent-ASV^® ^from admission to weaning or death. The rate and reason for stopping automation were recorded. Settings automatically selected, delivered ventilation, respiratory mechanics and arterial blood gas results were collected once a day. Patients were categorized in different lung conditions: normal lung, ALI/ARDS, COPD. Analysis of variance compared the ventilation-days for each type of lung condition for active and passive breathing patients.

## Results

Patients (age 73 (64 to 79) years; SAPS II 56 (48 to 69)) were ventilated using IntelliVent-ASV^® ^to weaning or death (31%) for a median duration of 3.0 (2.0 to 7.0) days without any safety issue. The ventilation controller was deactivated in two patients because of high PaCO_2_-ETCO_2 _gradient. Oxygenation controller was deactivated in seven patients for 1 day because of a poor SpO_2 _signal. In passive and active ventilation-days, minute volume, VT/PBW, respiratory rate, FiO_2_, and PEEP were statistically different based on lung condition. In passive ALI/ARDS ventilation-days, VT/PBW was significantly lower (7.5 (6.9 to 7.9) ml/kg) than passive normal lung (8.1 (7.3 to 8.9) ml/kg; *P *< 0.05) and passive COPD patients (9.9 (8.3 to 11.1) ml/kg; *P *< 0.05). In passive ALI/ARDS ventilation-days, FiO_2 _and PEEP were statistically higher than passive normal lung (35 (33 to 47)% vs. 30 (30 to 31)% and 11 (8 to 13) cmH_2_O vs. 5 (5 to 6) cmH_2_O, respectively; *P *< 0.05). In active normal lung ventilation-days, VT/PBW was not different (8.4 (7.8 to 9.1) ml/kg) than in active ALI/ARDS (8.1 (7.5 to 9.3) ml/kg), and in active COPD (9.3 (8.6 to 11.6) ml/kg). In active ALI/ARDS and COPD ventilation-days, PEEP was significantly higher than active normal lung (8 (5 to 10) cmH_2_O, 7 (5 to 10) cmH_2_O, and 5 (5 to 5) cm H_2_O, respectively; *P *< 0.05).

## Conclusion

IntelliVent-ASV^® ^can be used safely in long-term ventilated ICU patients and selects automatically different ventilation and oxygenation settings according to the lung condition, especially for passive breathing patients.

